# Long non-coding RNA anti-differentiation non-coding RNA affects proliferation, invasion, and migration of breast cancer cells by targeting miR-331

**DOI:** 10.1080/21655979.2021.2005989

**Published:** 2021-12-10

**Authors:** Chaoyu Jiang, Xianbiao Shi, Dandan Yi, Ru Wang, Fazhan Xu, Wenxian Guan, Jianfeng Sang

**Affiliations:** Department of General Surgery, Affiliated Drum Tower Hospital, Medical School of Nanjing University, Zhongshan Road, Nanjing, Jiangsu Province, China

**Keywords:** Anti-differentiation non-coding rna, breast cancer, invasion, migration, miR-331, proliferation

## Abstract

We aimed to evaluate the effects of long-chain non-coding RNA (lncRNA) anti-differentiation non-coding RNA (ANCR) on the proliferation, invasion, and migration of breast cancer cells by targeting miR-331. Forty-eight breast cancer and paracancerous tissue samples were collected. LncRNA ANCR expressions in breast cancer and adjacent tissues, human breast cancer cells and mammary epithelial cells, and miR-331 expressions in interfering cell line MDA-MB-231 (MCF-7)-shANCR, negative control MDA-MB-231 (MCF-7)-shNC and blank control MDA-MB-231 (MCF-7) were detected by real-time quantitative PCR. The correlations between lncRNA ANCR expression and clinicopathological characteristics were analyzed. Cell proliferation was detected by MTT and colony formation assays. Invasion and migration were tested by Transwell and scratch assays, respectively. The targeting relationship between ANCR and miR-331 was analyzed using the TargetScan database, and their interaction was studied using a dual-luciferase reporter assay. The expression of lncRNA ANCR in breast cancer tissue was significantly lower than that in adjacent normal tissue (p < 0.05). LncRNA ANCR was lowly expressed in various human breast cancer cell lines, being lowest in high-metastatic cell line (MDA-MB-231HM) (p < 0.05). Silencing lncRNA ANCR significantly enhanced the proliferation and invasion capacities of breast cancer cells, and promoted their tumor formation abilities in nude mice (p < 0.05). ANCR bound miR-331 targetedly, and the former negatively regulated the expression of the latter. LncRNA ANCR is lowly expressed upon breast cancer, and inhibits cell proliferation, invasion, and migration *in vitro* and *in vivo*. LncRNA ANCR exerts antitumor effects by targetedly binding miR-331 and then inhibiting its expression.

## Introduction

Breast cancer is one of the most common malignant tumors and also the second most dangerous killer among females worldwide. Many factors are potentially linked to the onset of breast cancer, including age, weight, use of hormones, smoking, etc. [1,2]. In China, the number of women with breast cancer is also increasing annually [3].

Many long non-coding RNAs (lncRNAs) have been discovered, and their associations with tumors have attracted widespread attention [4]. LncRNA is a subclass of non-coding RNA, which is generally longer than 200 nt, with a relatively conserved secondary structure but without protein coding ability. LncRNA has high tissue and cell specificities in life activities, and can influence biological behaviors by regulating the expression levels of mRNAs and proteins [5,6]. In recent years, lncRNA has been verified to play regulatory roles in signaling pathways such as tumor cell cycle, invasion, migration, and resistance to chemotherapy [7]. As a member of the lncRNA family, anti-differentiation noncoding RNA (ANCR) has a length of 855 nt, which was discovered by Kretz et al. in 2012 using transcriptome sequencing and tiling array [8]. ANCR is located on the human chromosome 4 between USP46 and ERVMER34-1 genes. There are three exons and two introns in the ANCR genomic region. Each of these two introns can produce a microRNA (MIR4449) and a snoRNA (SNORN26) which, however, do not exist in the final mature ANCR transcript. ANCR can inhibit the differentiation of epidermal progenitor cells, during which its expression is significantly down-regulated. Nevertheless, knocking down ANCR can significantly promote the differentiation and expressions of some genes, but the detailed molecular mechanisms remain unclear. Recently, ANCR has been proven to participate in regulating differentiation during the onset and progression of various diseases [9]. Although many non-coding RNAs, tumor suppressor genes, oncogenes, and protein factors are involved in the onset and progression of breast cancer, the role of ANCR has seldom been studied.

Thereby motivated, the lncRNA ANCR and miR-331 expressions in breast cancer and adjacent tissues were measured using real-time quantitative PCR (RT-PCR), and the associations with the clinicopathological parameters of patients were explored. The lncRNA ANCR expression in breast cancer cell line MDA-MB-231 was down-regulated, aiming to evaluate the effects on cell proliferation, invasion, and migration.

## Materials and Methods

### Tissue, cells, reagents, and apparatus

Forty-eight breast cancer tissue samples and corresponding paracancerous tissue samples were obtained from patients who underwent surgery in our hospital from January 2018 to January 2019. The patients were aged 36–65 years old, with the median of 47. All patients agreed to this study and signed informed consent, and the samples were collected after review and approval by the ethics committee of our hospital.

Human breast cancer cells BT474, MCF7, T47D, MDA-MB-436, MDA-MB-231, human breast cancer high-metastatic cell line MDA-MB-231HM, human immortalized mammary epithelial cell line MCF10A, and human embryonic kidney cell line HEK293T were purchased from the American Type Culture Collection (USA). Stably transfected cell line MDA-MB-231-shANCR, negative control MDA-MB-231-shNC, ANCR, GAPDH, and miR-331 primers were provided by Shanghai GenePharma Co., Ltd. (China). DMEM/F12 medium (D8900), DMEM (D777), 1640 medium (R6504), and L15 medium (L4386) were bought from Sigma (USA). SYBR Green RT-PCR kit (QPK-201) was purchased from Toyobo (Japan). Dual-luciferase reporter assay kit (RG027) was obtained from Beyotime Institute of Biotechnology Co., Ltd. (China). Trizol reagent (9009) was provided by TaKaRa (Japan). Matrigel (356,234) was purchased from BD (USA). The main apparatus included Sanyo MCO-15AC cell culture incubator (Japan), Nikon Ti-U/Ti-s inverted fluorescence microscope (Japan), Eppendorf 5810 R high-speed centrifuge (USA), and Roche R480 RT-PCR system (USA).

### Detection of lncRNA ANCR and miR-331 expressions by RT-PCR

Total RNA was extracted by Trizol reagent from the collected breast cancer tissue and corresponding paracancerous tissue samples, human breast cancer cells, MDA-MB-231HM, MCF10A, MDA-MB-231-shANCR and MDA-MB-231-shNC cells, and reverse-transcribed. The resulting cDNA was used as a template for RT-PCR to detect the expression of lncRNA ANCR, with GAPDH as the internal reference. Each sample was tested in triplicate independently [10].

### Detection of cell proliferation by MTT assay and colony formation assay

Cells were added to 96-well plates at a density of 500–1000/well, gently mixed, placed in a 37°C incubator to continue culture, added 20 μl of MTT solution per well, and incubated in the dark at 37°C for 4 h. After the liquid in each well was discarded, the residue was added 100 μl of DMSO per well, and shaken quickly on a 37°C shaker for 15 min to fully dissolve the crystals. Finally, the optical density (OD) of each well was measured by a microplate reader at 492 nm. The stably transfected cells were cultured in 6-well plates at the density of 5 × 10^3^/well, and fresh medium was replaced every other week. After 2 weeks, the cells were stained with Coomassie blue, and the colonies were counted under a microscope [11].

### Detection of cell invasion by Transwell assay

The medium was replaced with a serum-free medium 12 h before the test, and 40 μl of Matrigel was added to each Transwell chamber. The cells were digested and washed twice with 1× PBS, and then 500 μl of complete medium was added to 24-well plates. Afterward, 5 × 10^5^ cells were collected for resuspension, and 200–250 μl of the suspension was added to each Transwell chamber. After 24 h of culture, 500 μl of 0.1% crystal violet staining solution prepared with methanol and diluted with PBS was added to stain the cells in the dark at room temperature for 15 min. Then, the cells were rinsed with PBS, and the inside of Transwell chamber was wiped with a cotton swab. The chamber was inverted, dried in air, observed under an inverted fluorescence microscope, photographed, and counted [12].

### Detection of cell migration by scratch assay

MDA-MB-23, MDA-MB-231-shANCR, and MDA-MB-231-shNC cells were evenly inoculated into 6-well plates at the density of 5 × 10^5^, scratched with a 10 μl pipette tip, added PBS to rinse the residue, added medium containing 1% serum, observed under a microscope, photographed, and marked, with the time recorded as 0 h. Subsequently, the cells were cultured in a 37°C incubator with 5% CO_2_. The cell movement was observed under an inverted microscope and photographed at 12 h, 24 h, and 48 h, respectively [13].

### Establishment of nude mouse xenograft model

Four-week-old female BALB/c nude mice were fed under specific pathogen-free conditions. MDA-MB-231-siANCR and MDA-MB-231-siNC cells were injected into the axilla at a concentration of 1 × 10^6^. Feeding was continued for 5 weeks, and then the volume and growth of the formed tumors were measured. Tumor volume = 1/2 (shortest diameter)^2^ × (longest diameter).

### Dual luciferase reporter assay

Using normal human genomic DNA as a template, lncRNA ANCR containing miR-331 and lncRNA ANCR complementary sites was amplified by PCR and constructed into luciferase reporter vector psi-CHECK, which was referred to as lncRNA ANCR-wild. Meanwhile, the mutant recombinant plasmid lncRNA ANCR-mutant was constructed. HEK293T cells were placed on a 24-well plate at a density of about 30% and co-transfected by Renilla luciferase plasmid, miR-331, and its negative control together with lncRNA ANCR-wild and mutant lncRNA ANCR-mutant, respectively. After 24 h, the luciferase activity was measured according to the instructions of dual-luciferase assay kit, and the ratio of firefly luciferase activity/Renilla luciferase activity was employed as the reporter gene activity [14].

## RESULTS

### LncRNA ANCR expressions in breast cancer tissues and cells

**Figure 1.**
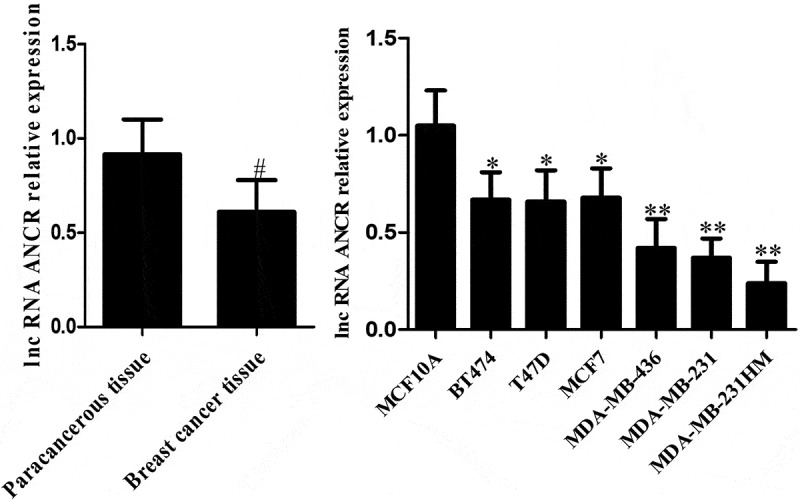
LncRNA ANCR expressions in breast cancer tissues (n = 48) and cells (n = 3). **Compared with MCF10A cells, p < 0.001; #compared with paracancerous tissue, p < 0.05

**Figure 2.**
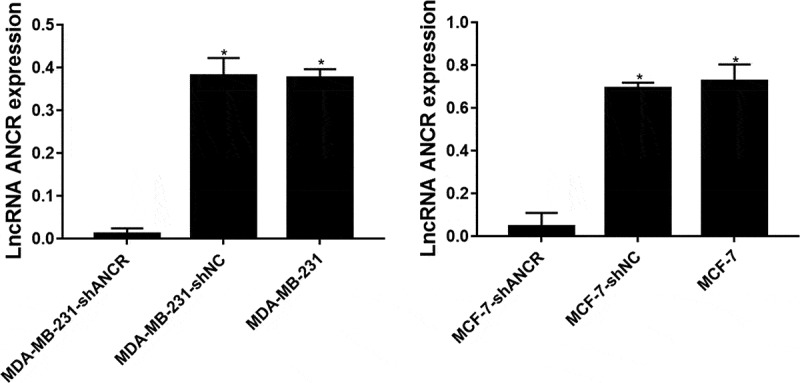
LncRNA ANCR expressions in cells after transfection. All experiments were performed in triplicate independently. *Compared with MDA-MB-231 (MCF-7)-shANCR cells, p < 0.05

ANCR, which is located on the human chromosome 4, was reported for the first time in regulating the differentiation of progenitor cells. The possibility of its non-coding transcript is 7.64 × 10^90^ times that of protein-coding transcripts [15]. The expression level of lncRNA ANCR in breast cancer tissue was significantly lower than that in adjacent normal tissue (p = 0.024). Compared with mammary epithelial cells MCF10A, lncRNA ANCR was lowly expressed in various human breast cancer cell lines, being lowest in high-metastatic cell line (MDA-MB-231HM) (p < 0.05) (Figure 1). Therefore, lncRNA ANCR probably inhibited breast cancer and was negatively correlated with its migration. The expression of lncRNA ANCR in MDA-MB-231 (MCF-7)-shANCR cells was significantly lower than those of MDA-MB-231 (MCF-7)-shNC and MDA-MB-231 (MCF-7) cells, indicating that stably transfected cell lines had been successfully constructed (Figure 2).

ANCR is highly expressed in liver cancer tissues and cells and positively correlated with tumor size, degree of differentiation, TNM stage, and portal vein tumor thrombi [16]. Nevertheless, its role in breast cancer has seldom been referred to. LncRNA ANCR expression was considered high based on 70% of that of total cases. The expression level of lncRNA ANCR was not correlated to age (p > 0.05). Such expression significantly decreased with increasing tumor volume and clinical stage, as well as in the case of lymph node metastasis, also with significant differences among various subtypes (p < 0.05) (Table 1).

**Table 1.** Correlations between lncRNA ANCR expression and clinicopathological characteristics of patients with breast cancer

Characteristic	High expression (n = 15) (%)	Low expression (n = 33) (%)	χ^2^	p
Age (year)			0.257	0.613
≤40	7	18		
>40	8	15		
Tumor volume (cm^3^)			8.394	0.004
≤2	9	6		
>2	6	27		
Clinical stage			Fisher’s	0.025
I	6	5		
II	5	4		
III	4	21		
IV	0	3		
Lymph node metastasis			5.610	0.018
Yes	5	23		
No	10	10		
Subtype			8.097	0.044
Luminal A	2	8		
Luminal B	8	5		
HER-2 overexpression	2	12		
Basal-like	3	8		

### Effect of lncRNA ANCR on MDA-MB-231 cell proliferation

**Figure 3.**
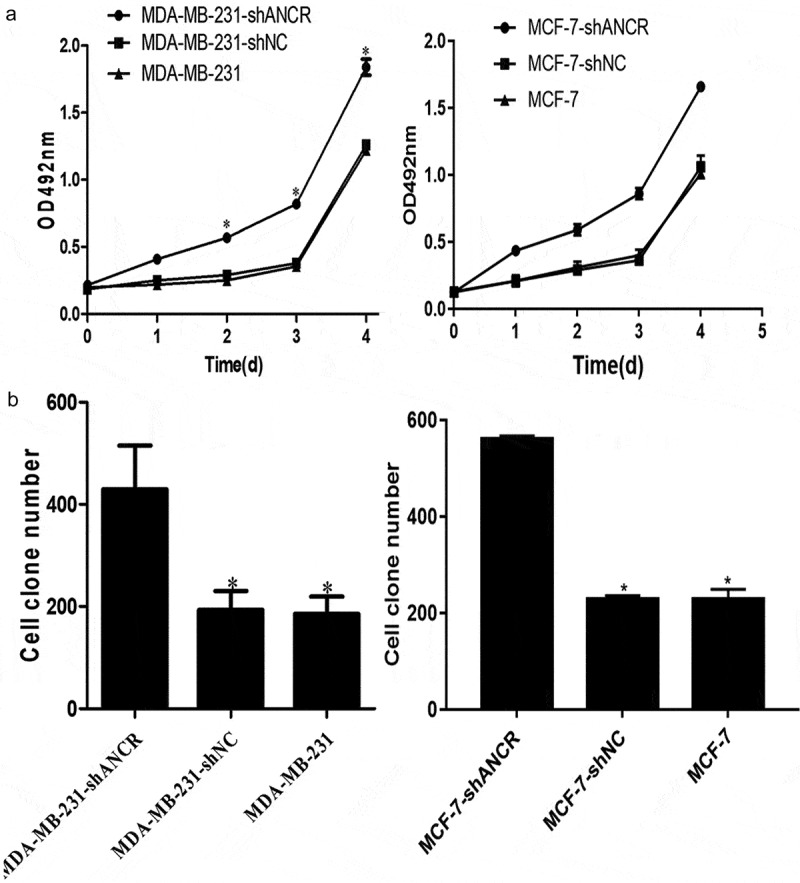
Effect of lncRNA ANCR on MDA-MB-231 (MCF-7) cell proliferation. All experiments were performed in triplicate independently. A: MTT assay results; B: colony formation assay results

ANCR plays essential roles in the proliferation, migration, invasion, epithelial–mesenchymal transition, and metastasis of various tumors [17,18]. MTT assay showed that silencing lncRNA ANCR significantly enhanced OD_492 nm_ of MDA-MB-231 cells (p < 0.05) (Figure 3(a)). Colony formation assay exhibited that silencing lncRNA ANCR significantly increased the colony number of MDA-MB-231 (MCF-7) cells (p < 0.05) (Figure 3(b)). Thus, breast cancer cell proliferation was promoted by silencing lncRNA ANCR.

### Effect of lncRNA ANCR on MDA-MB-231 cell invasion and migration

**Figure 4.**
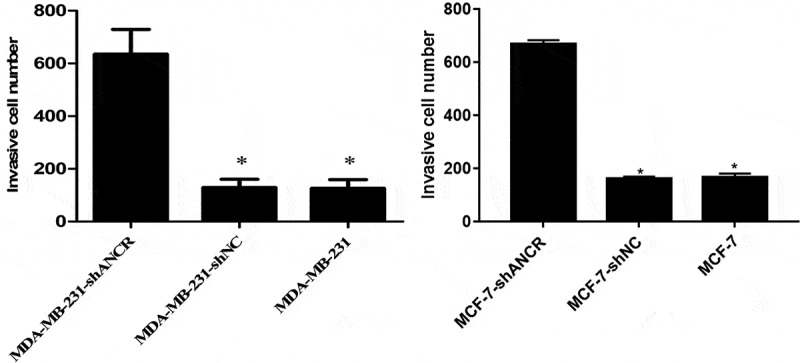
Effect of lncRNA ANCR on MDA-MB-231 (MCF-7) cell invasion. All experiments were performed in triplicate independently. Compared with MDA-MB-231-shANCR cells, *p < 0.05

Transwell assay revealed that silencing lncRNA ANCR significantly increased the number of MDA-MB-231 (MCF-7) cells penetrating Matrigel (p < 0.05) (Figure 4), indicating evidently enhanced invasion ability.

**Figure 5.**
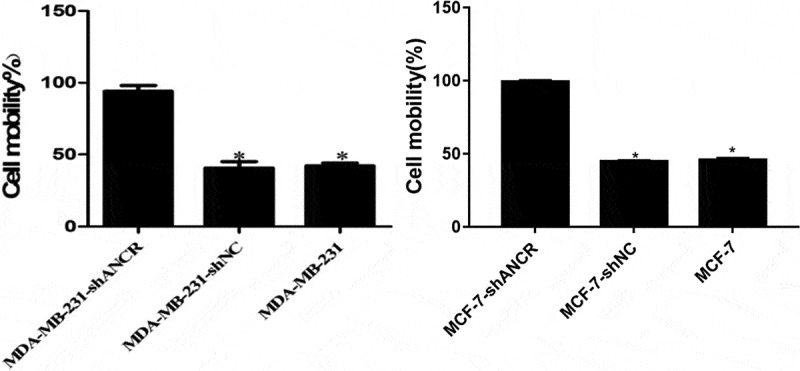
Effect of lncRNA ANCR on MDA-MB-231 (MCF-7) cell migration. All experiments were performed in triplicate independently. Compared with MDA-MB-231-shANCR cells, *p < 0.05

Scratch assay showed that silencing lncRNA ANCR significantly accelerated cell wound healing (p < 0.05) (Figure 5), suggesting significantly elevated migration capacity.

### Effect of lncRNA ANCR on tumor formation in nude mice

**Figure 6.**
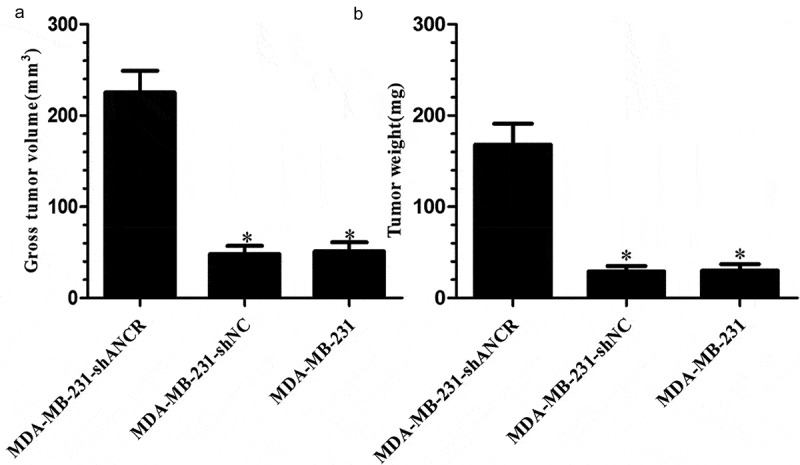
Effect of lncRNA ANCR on tumor formation in nude mice (n = 8). A: Tumor volumes; B: tumor weights. Compared with MDA-MB-231-shANCR group, *p < 0.05

Five weeks after xenografting, the MDA-MB-231-shANCR group had significantly larger tumor weight and volume than those of other groups (p < 0.05) (Figure 6). Accordingly, knocking down lncRNA ANCR facilitated tumor formation in nude mice.

### Relationship between lncRNA ANCR and miR-331

**Figure 7.**
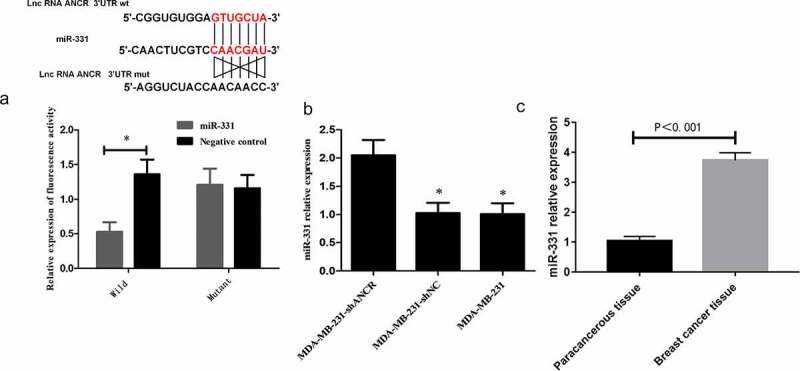
Relationship between lncRNA ANCR and miR-331. A: Dual luciferase reporter assay results; B: RT-PCR results. Compared with MDA-MB-231-shANCR group, *p < 0.05; C: miR-331 expressions in breast cancer and adjacent tissues (n = 48). All experiments were performed in triplicate independently

LncRNA can interact with protein or microRNA sponge to regulate the expressions of target molecules, thereby participating in tumor metastasis [19,20]. ANCR may play a crucial role in regulating the stability of its binding proteins by interacting with them. However, the regulatory mechanism of ANCR in breast cancer is still unclear. The TargetScan database (http://www.targetscan.org/vert_72/) exhibited that there may be a targeted regulatory relationship between ANCR and miR-331 [21]. Dual-luciferase reporter assay presented that after miR-331 transfection, the activity of wild-type ANCR was significantly attenuated (p < 0.05), whereas that of mutant ANCE remained almost unchanged (p > 0.05) (Figure 7(a)), indicating that ANCR bound miR-331 targetedly. RT-PCR revealed that after lncRNA ANCR was silenced, the miR-331 expression level was significantly up-regulated (p < 0.05) (Figure 7(b)). The miR-331 expression in breast cancer tissue was significantly higher than that in adjacent tissue (p < 0.05) (Figure 7(c)). LncRNA ANCR and miR-331 expressions in clinical tissue samples were subjected to Pearson’s correlation analysis, showing a significant negative correlation (r = −0.729, p < 0.001). Hence, lncRNA ANCR negatively regulated miR-331.

## DISCUSSION

LncRNA ANCR was discovered in 2012 by Kretz et al. using tiling arrays technology and transcriptome sequencing [8,9]. It contains 855 nucleotides and is located on the chromosome 4 upstream of human USP46 gene, producing mIR4449 in the middle of two introns of the genome and SNORNA26, but not in the final mature transcript [22,23]. During osteoblast differentiation, ANCR binds EZH2 to increase H3K27me3 in the promoter region of Runx2 gene, thereby inhibiting Runx2 transcription [24–26]. The expression of lncRNA ANCR significantly decreased during the differentiation of epidermal progenitor cells. After lncRNA ANCR was silenced, the differentiation ability of progenitor cells increased, and the genes involved in differentiation were also expressed in large amounts. ANCR inhibited the differentiation of progenitor cells [27]. In addition, down-regulating lncRNA ANCR regulates EZH2 expression and inhibits the invasion and migration of colorectal cancer [18]. In breast cancer cells, lncRNA ANCR binds EZH2 and also promotes the binding of CDK1 kinase to EZH2, which increases the phosphorylation of threonine Thr-345 and Thr-487 in EZH2, ultimately leading to the ubiquitination of EZH2 [17,28]. This study first confirmed the low expression of lncRNA ANCR in breast cancer tissues and cell lines by RT-PCR, and the lowest expression in high-metastatic MDA-MB-231HM cells. LncRNA ANCR has an inhibitory effect and a negative correlation with cell migration. Given that the patients with lymph node metastasis had larger tumor volume, higher clinical stage, and lower expression level of lncRNA ANCR, ANCR may be a tumor suppressor. MTT and colony formation assays showed that the proliferation of MDA-MB-231 cells significantly increased after silencing of lncRNA ANCR, indicating that ANCR exerted an inhibitory effect on the proliferation of breast cancer cells. Transwell assay showed that the invasive ability of MDA-MB-231 cells significantly increased after silencing lncRNA ANCR, indicating that ANCR can reduce the invasion of breast cancer cells. Scratch assay showed that the migration ability of MDA-MB-231 cells significantly increased after silencing lncRNA ANCR, indicating that ANCR can inhibit the migration of breast cancer cells. The nude mouse model of tumor formation showed that knockdown of lncRNA ANCR promoted the growth of transplanted tumors in mice. Thus, lncRNA ANCR can inhibit the proliferation of breast cancer cells *in vivo*. Collectively, lncRNA ANCR plays an important regulatory role in the development and progression of breast cancer, as being a potential marker for treatment and prognosis.

As non-coding single-stranded small RNAs of 18–25 nt in length in eukaryotes, miRNAs directly bind the 3ʹ non-transcribed region of a specific target messenger RNA. They promote the degradation of target RNA or the inhibition of translation, thereby regulating the expression of target gene. A wide variety of miRNA family members are closely related to the development of many human diseases and tumors [29,30]. MiR-331 gene is located on 12q22n of human chromosome. Related studies have confirmed that miR-331 can inhibit prostate, gastric, colorectal, and cervical cancers, but promote the proliferation and metastasis of liver and promote cancer cells. In lymphocytic leukemia, miR-331 is abnormally expressed and involved in cell proliferation and migration [31–33]. Accumulating evidence has proven that lncRNA ANCR works as a tumor suppressor gene in the progression of breast cancer. However, whether miR-331 is involved in the development of lncRNA ANCR regulation of breast cancer remains largely unknown. LncRNA has multi-directional regulatory effects on different cells and thus dominates in many human diseases. Studies have confirmed a new regulatory mechanism between mRNA and lncRNA, i.e. the interaction between them and their common miRNA response elements. LncRNA may function as a competitive endogenous RNA, adsorbing miRNAs and regulating their targets, thereby further facilitating post-transcriptional regulation. In this study, the TargetScan database was used to predict the target gene of lncRNA ANCR. It was found that there was a binding site between lncRNA ANCR and miR-331, suggesting that miR-331 can be used as a downstream target gene of lncRNA ANCR. Moreover, the luciferase reporter gene assay exhibited that ncRNA ANCR and miR-331 formed a complementary base pair, which further confirmed the targeting relationship between them. RT-PCR revealed that miR-331 expression was significantly up-regulated after silencing lncRNA ANCR in breast cancer cells, confirming that lncRNA ANCR exerted a negative regulatory effect on miR-331. Therefore, lncRNA ANCR may work as a tumor suppressor by specifically binding miR-331 and inhibiting its expression in breast cancer.

## Conclusion

In summary, lncRNA ANCR is lowly expressed in breast cancer and can inhibit the proliferation, invasion, and migration of breast cancer cells *in vitro* and *in vivo*. It can bind miR-331 and inhibit its expression to exert a tumor-suppressing effect.

## Highlights

- LncRNA ANCR is lowly expressed upon breast cancer.
- LncRNA ANCR inhibits cell proliferation, invasion, and migration.
- It exerts antitumor effects by binding miR-331 and inhibiting its expression.
